# Correlation Between Neuroimaging and Neuropathologic Findings in Pediatric ECMO Patients

**DOI:** 10.21203/rs.3.rs-8745399/v1

**Published:** 2026-02-09

**Authors:** Margherita Tabet, Bill Zhang, Chasity Custer, Michael Craig Morriss, Sumit Singh, Aliya Abioye, Ethan Sanford, Lakshmi Raman, Veena Rajaram, David R. Busch

**Affiliations:** The University of Texas Southwestern Medical Center; The University of Texas Southwestern Medical Center; The University of Texas Southwestern Medical Center; The University of Texas Southwestern Medical Center; The University of Texas Southwestern Medical Center; The University of Texas Southwestern Medical Center; The University of Texas Southwestern Medical Center; The University of Texas Southwestern Medical Center; The University of Texas Southwestern Medical Center; The University of Texas Southwestern Medical Center

**Keywords:** Pediatrics, Brain Injuries, Neuropathology, Neuroimaging, Extracorporeal Membrane Oxygenation, Critical Care, Neurodiagnostic Techniques

## Abstract

**Objective:**

Neurologic complications are a leading cause of morbidity and mortality in children supported with extracorporeal membrane oxygenation (ECMO). Antemortem neuroimaging is limited, while postmortem neuropathology provides definitive but rarely available assessment. Correlating imaging with pathology may improve diagnostic accuracy and monitoring strategies.

**Design:**

We performed a retrospective, single-center cohort study of neonates and children (0–18 years) supported with ECMO (2009–2024) who underwent cerebral autopsy. Standardized neuropathologic sampling across six brain regions classified injuries as mild, moderate, or severe. Antemortem imaging (CT or head ultrasound [HUS]) within seven days of death was reviewed by a blinded neuroradiologist and scored using a Neurologic Injury Severity (NIS) framework. Imaging-pathology correlation was assessed in subjects with both datasets.

**Measurements and Main Results:**

Fifty-four patients underwent neuropathologic evaluation; 26 had qualifying neuroimaging. Neuropathology revealed frequent ischemic and hemorrhagic injuries, most commonly in the frontal lobes, deep grey matter, and temporal lobes. Severe injury was more frequent in venoarterial ECMO and in infants/toddlers. NIS scores strongly correlated with global and regional histopathologic severity, particularly in frontal, temporal, and deep grey regions. Discordance was observed in parieto-occipital, pontine, and cerebellar regions, where immunohistochemistry (GFAP, CD68) detected subtle injuries not visible on H&E. Severe pathology consistently corresponded to NIS ≥10, while mild pathology aligned with low scores and limited involvement.

**Conclusions:**

Structured imaging severity scoring, especially with CT, correlates strongly with neuropathology in pediatric ECMO decedents. Findings support NIS scoring as a surrogate for underlying pathology, while underscoring the need for refined histologic methods and adjunctive neuromonitoring to optimize neurologic surveillance.

## Introduction

Extracorporeal membrane oxygenation (ECMO) is a life-support intervention for neonates and children with severe cardiac or respiratory failure, with more than 90,000 pediatric cases treated globally.^1^ Pediatric survival has improved, now averaging around 54% across indications including extracorporeal cardiopulmonary resuscitation (ECPR), cardiac, and pulmonary support.^2^ Despite these advances, neurologic complications remain a major cause of morbidity and mortality.^3^ Acute brain injuries (ABIs), such as hypoxic-ischemic injury, ischemic stroke, and intracranial hemorrhage, are particularly devastating and often difficult to detect during ECMO.^4^ Neurologic assessment is hindered by sedation, neuromuscular blockade, and the risks of patient transport. Routine neuroimaging is therefore reserved for high-suspicion scenarios such as pupillary abnormalities, new focal EEG findings, unexplained hemoglobin drops, or acute neurologic deterioration.^5–7^ In some cases, imaging informs discussions about withdrawal of life-sustaining therapies. The most common modalities are head ultrasound (HUS) and computed tomography (CT), while magnetic resonance imaging (MRI) remains rare due to technical and safety challenges.^8^ Subtle or early injuries, however, are often missed.^9^ Comprehensive postmortem neuropathologic assessments are also infrequent, limiting correlation between imaging and histopathology.^10^ In adult ECMO, postmortem studies consistently reveal high ABI rates, especially hypoxic-ischemic injury linked to impaired autoregulation and altered perfusion in venoarterial ECMO (VA-ECMO).^11,12^ Many of these injuries are underrecognized clinically.^13^ Pediatric studies show similar patterns, with ischemic and hemorrhagic injuries exacerbated by the vulnerabilities of the immature brain.^3^ Neuropathology provides critical insights, as histologic evaluation often reveals injuries undetected by imaging.^14–17^ These discrepancies highlight the need for improved diagnostic strategies.^18^

This study aims to characterize the types and distribution of neurologic injury in pediatric ECMO decedents and compare antemortem neuroimaging findings with postmortem neuropathology, in order to determine how well clinically obtained imaging reflects underlying tissue-level injury and to identify diagnostic limitations relevant to neuromonitoring strategies.

## Methods

### Study Design and Oversight:

This observational, single-center cohort study was conducted at Children’s Medical Center, Dallas, in accordance with the Committee for the Protection of Human Subjects at the University of Texas Southwestern Medical Center. The protocol (STU-2022–0723) was approved by the institutional review board on 09/12/2024. Informed consent was waived due to the retrospective design.

### Study Population

Subjects were neonates and children (0–18 years) supported with ECMO between January 1, 2009, and August 1, 2024. Inclusion required death during ECMO or within three days of decannulation, with cerebral autopsy performed. For the imaging sub-analysis, eligible patients also had neuroimaging within seven days of death.

### Demographic and Clinical Data Collection

Demographic and baseline clinical data, including sex, race, ethnicity, height, weight, primary diagnosis, ECMO type (veno-venous [VV] or veno-arterial [VA]), oxygenator, and ECPR status, were abstracted from electronic health records and autopsy reports. Continuous variables were reported as means ± standard deviation, and categorical variables as counts and percentages.

### Neuropathologic Examination:

Postmortem examinations were performed by a board-certified neuropathologist blinded to imaging and clinical data. Gross and microscopic examinations used hematoxylin and eosin (H&E). When no obvious injury was observed, immunohistochemistry with GFAP and CD68 was performed to detect gliosis and microglial activation. Histological sampling was standardized across six brain regions: frontal lobes, deep grey matter, temporal lobes, parieto-occipital lobes, pons, and cerebellum.

### Contextualized Classification of Neuropathologic Injury

Neuropathologic injuries were classified into mild, moderate, and severe categories based on histologic findings. Mild injury was defined as limited or nonspecific abnormalities, such as absent white matter injury, disorganized myelination, or slight ventricular dilation. These were usually scattered and restricted to one or two regions, without overt parenchymal damage. Moderate injury referred to more focal or localized abnormalities, such as white matter pallor, focal hemorrhage, ischemic changes, or neuronal necrosis, often spanning multiple regions but not reflecting diffuse involvement. Severe injury encompassed widespread, multifocal pathology, including diffuse ischemia, multiple hemorrhages, subdural hemorrhage with necrosis, cortical edema, and necrotic infarcts. These findings often involved both cortical and subcortical regions, including brainstem and cerebellum, and reflected diffuse cerebral injury. Each finding was mapped across brain regions, enabling structured quantification of anatomical burden **(Supplemental digital content-Table 1).**

#### Neuroimaging Acquisition, Analysis, and Neurologic Injury Severity (NIS) Score Determination

Antemortem imaging included CT and HUS. When both were available, CT was prioritized. All studies were reviewed by a blinded neuroradiologist. Abnormalities were classified into hemorrhagic, ischemic, or ventricular dilation categories and assigned severity scores. NIS scores were calculated for each subject, based on radiologic scoring frameworks adapted to pediatric ECMO.^19,20^ Categories were defined as mild (0–2), moderate (3–10), and severe (≥10), aligned with neurologic outcomes measured by the Pediatric Cerebral Performance Category (PCPC).^21^

### Sub-analysis: Neuropathology and Neuroimaging Correlation

Of the 54 patients with neuropathology, 26 had qualifying neuroimaging for correlation. For each, brain regions were graded (none, mild, moderate, severe) by histopathology and compared against NIS scores.

## Results

### Demographics

Fifty-four ECMO patients underwent neuropathologic evaluation (29 males, 25 females). Most were White (70%) or Black (28%), and 72% were non-Hispanic. The mean weight was 20.8 kg, and mean height was 92.7 cm. Age groups included 9 neonates, 17 infants/toddlers, 15 children, and 13 adolescents. Pulmonary diagnoses accounted for 44% of cases, cardiac for 17%, and ECPR for 39%. VA ECMO supported 56% of patients, while VV ECMO supported 44%. The most frequent oxygenator was the Quadrox i (56%) (Table 1). The proximate cause of death was cardiorespiratory failure in the majority of cases, with withdrawal of life-sustaining therapy due to multisystem organ failure in others; primary neurologic injury was rarely the sole cause of death.

### Correlation of Gross and Microscopic Neuropathologic Findings by Brain Region

**Figure 1:**
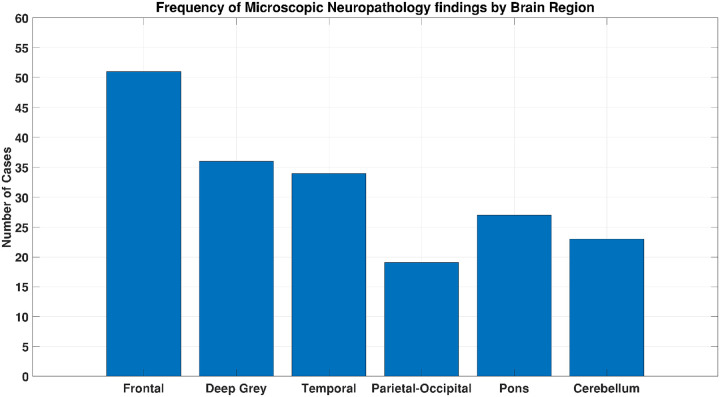
Frequency of microscopic neuropathology findings by brain region.

Neuropathology revealed diverse injury patterns involving multiple brain regions. Frontal lobes showed gliosis, red neurons, and infarcts in most cases. Deep grey matter demonstrated hypoxic changes such as reactive gliosis and edema. Temporal lobes, particularly hippocampi, displayed ischemic necrosis. Parieto-occipital regions showed selective white matter changes. Pons and cerebellum occasionally exhibited infarcts, inflammatory injury, and Purkinje cell necrosis. Microscopy provided greater detail than gross pathology, often revealing injuries invisible macroscopically (Figure 1).

### Region-specific Neuropathological patterns across ECMO modalities and Age groups

**Figure 2:**
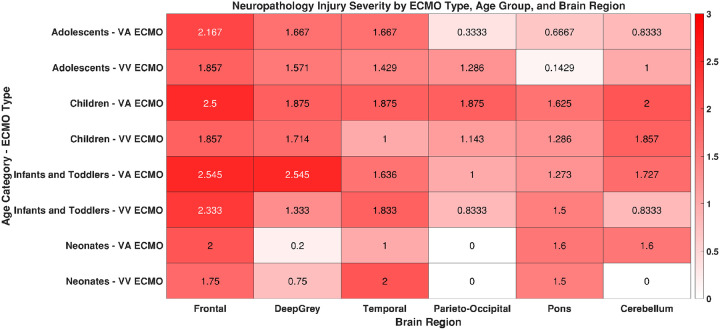
Heatmap of Neuropathological Injury Severity by Brain Region, ECMO Modality, and Age.

Neuropathologic patterns varied by age and ECMO modality (Figure 2, **Supplemental digital content-Table 2).** In adolescents, VA ECMO was associated with moderate-to-severe injury in frontal, deep grey, and temporal regions, with milder pontine and cerebellar involvement. VV ECMO produced similar but more localized injury. In children, VA ECMO caused widespread severe injury, while VV ECMO resulted in milder but still diffuse changes. Infants and toddlers had severe frontal and deep grey injuries with both modalities, with additional moderate injuries in temporal, parieto-occipital, pontine, and cerebellar regions. Neonates showed moderate injury in frontal, temporal, and infratentorial regions for both VA and VV ECMO, though VV ECMO appeared to spare the cerebellum and parieto-occipital lobes. Cerebellar involvement was otherwise rare except in VA ECMO-treated neonates and children.

### Correlation between NIS and Gross Neuropathology Findings

Of the 54 pediatric ECMO decedents with available neuropathologic data, 26 met inclusion criteria for the imaging-pathology correlation analysis. These subjects underwent neuroimaging, either HUS or CT, within seven days prior to death, with image quality sufficient for NIS scoring. When both modalities were available, CT was prioritized due to its superior spatial resolution and diagnostic detail. Neuroimaging scores were categorized to reflect injury severity: mild (NIS 0–3), moderate (NIS 4–9), and severe (NIS ≥10). A total of 15 subjects (58%) were classified as having severe injury, 3 (12%) as moderate, and 8 (31%) as mild. Gross neuropathologic findings correlated with these categories**(Supplemental digital content-Table 3).** Cases with severe NIS typically exhibited extensive or destructive patterns, including diffuse hypoxic-ischemic encephalopathy, organizing infarcts, and petechial hemorrhages. Moderate NIS cases showed more localized pathology such as ischemic changes or non-necrotizing meningoencephalitis. In contrast, mild NIS cases often demonstrated either subtle or no grossly appreciable brain injury. This distribution supports a strong correlation between higher NIS and the extent of gross pathological brain injury.

### Total NIS vs. Global Injury Severity

**Figure 3:**
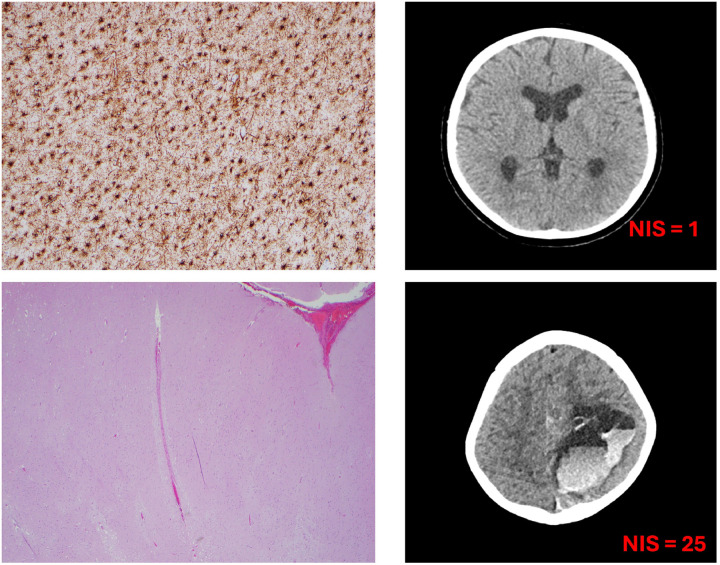
Histopathological and neuroimaging findings corresponding to Neurologic Injury Severity (NIS) scores. Top panel: NIS 1 (mild injury) characterized by mild patchy reactive gliosis in grey and white matter (left) and mild volume loss on imaging (right). Bottom panel: NIS 25 (severe injury) subarachnoid hemorrhage with ischemic parenchymal injury (left), with imaging evidence of large intracerebral, intraventricular and extra axial hemorrhages (right).

**Figure 4:**
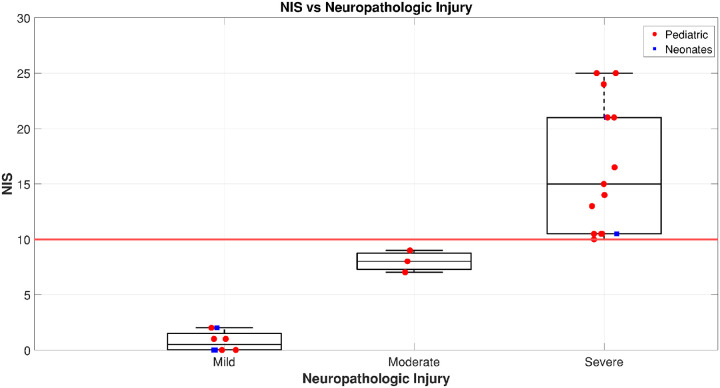
Box plot comparing total Neurologic Injury Severity (NIS) scores in subjects stratified by overall neuropathologic injury classification (mild, moderate, severe).

Subjects with severe injury demonstrated markedly higher NIS scores(Figure 3), with a wider interquartile range, reflecting both greater overall injury burden and substantial inter-individual variability. In contrast, subjects in the mild and moderate groups showed lower NIS values with relatively narrow IQRs, suggesting more consistent and less extensive injury patterns in these groups. Figure 4 compares total NIS scores across three neuropathologic injury categories: mild, moderate, and severe.

### Region-Specific Correlations

**Figure 5:**
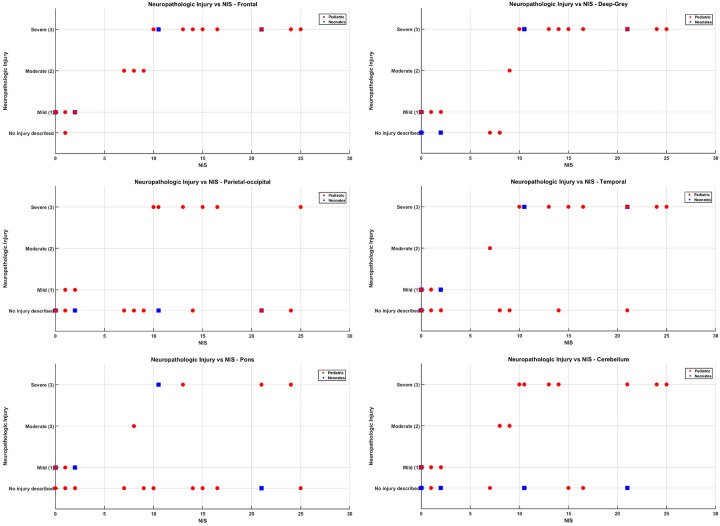
Region-specific correlation between neuropathologic injury level and Neurologic Injury Severity (NIS) score. Neonates are indicated with blue squares, while the remaining pediatric population is represented by black dots.

Visual inspection of region-specific scatter plots (Figure 5a–f), along with stratified analysis by injury level and age category, revealed consistent trends linking increasing histopathologic injury severity with higher Neurologic Injury Severity (NIS) scores across all six brain regions in a cohort of 26 subjects. These trends were particularly robust in older pediatric groups. The enhanced sensitivity of CT imaging to deeper brain structures likely contributed to stronger correlations, particularly in the deep grey matter and posterior regions. Age played a critical role in both the pattern of injury and the alignment between imaging and pathology.

- Frontal Lobes (Figure 5a): Severe injury (grade 3) was observed in 15 of 26 subjects, predominantly infants and toddlers (n=9), with additional cases in children (n=2), adolescents (n=2), and neonates (n=2). Mild injury (grade 1) was present in seven subjects, including three neonates, two infants and toddlers, one child and one adolescent. Subjects with severe frontal injury exhibited NIS scores ≥10. Strong imaging-pathology concordance was most evident in infants and toddlers.
- Deep Grey Matter (Figure 5b): Severe injury was identified in 15 subjects, including 9 infants and toddlers, 2 children, 2 adolescents, and 2 neonates. Mild injury occurred in six subjects (including one neonate), while moderate injury was observed in one infant. This region showed one of the strongest correlations between injury level and NIS, likely due to CT’s enhanced sensitivity for deep structures.
- Parieto-Occipital Lobes (Figure 5c): Severe injury in the parieto-occipital region was identified in 7 subjects, predominantly among infants and toddlers (n=5), with one case each in children and adolescents. Interestingly, two infants and toddlers and one child exhibited moderate NIS scores despite the absence of detectable parieto-occipital injury on standard H&E micropathology. In one infant, however, CD68 and GFAP immunostaining revealed occasionally activated microglia and reactive gliosis in the white matter, suggesting a subtle parieto-occipital injury not captured by conventional histological methods. For the remaining cases with moderate NIS scores and no parieto-occipital injury, the neurological impairment is likely attributable to damage in other regions, specifically the frontal and/or deep grey matter, which can significantly impact function even when the parieto-occipital lobes appear unaffected. Notably, no moderate neuropathological injuries were recorded in this region.
- Temporal Lobes (Figure 5d): Thirteen subjects had severe injury (8 infants and toddlers, 2 children, 2 adolescents, 1 neonate). Mild injury was seen in four subjects, including two neonates and moderate injury was seen in one infant. A stepwise increase in NIS scores paralleled injury severity, demonstrating strong imaging-pathology agreement across age groups.
- Pons (Figure 5e): Severe pontine injury was observed in eight subjects, including five infants and toddlers, and one subject each from the adolescent, child, and neonate groups. One child exhibited moderate injury, while five subjects, three of whom were neonates, had mild injury. Notably, seven subjects (four infants and toddlers, and one each from the adolescent, child, and neonate groups) demonstrated severe NIS scores despite no described pontine injury on standard histopathology. Among these, five showed scattered perivascular macrophages and reactive gliosis on GFAP and CD68 immunostaining, suggesting subtle or diffuse injury not captured by routine methods. Additionally, two infants with no described pontine injury had moderate NIS scores; both exhibited mild perivascular macrophages, rare, activated microglia, and mild reactive gliosis. Two other infants with no reported pontine injury were classified as having mild injury; one of them showed mild perivascular macrophages, increased activated microglia in the white matter, and mild reactive gliosis on GFAP and CD68. These findings highlight the potential for immunohistochemical markers to reveal underlying pathology not evident on conventional staining.
- Cerebellum (Figure 5f): Severe cerebellar injury was identified in ten subjects, including seven infants and toddlers, two children, and one adolescent, all of whom had severe NIS scores. Moderate cerebellar injury was observed in one child and one infant, both corresponding with moderate NIS scores. Among five subjects with mild cerebellar injury, only two had mild NIS scores, while the remaining three had NIS scores of zero. Of these three, two showed scattered perivascular macrophages and mild reactive gliosis in the white matter, suggesting subtle pathology not captured by standard scoring. Additionally, nine subjects exhibited no micropathological evidence of cerebellar injury despite having NIS scores ranging from mild to severe. In these cases, neurological impairment is likely attributable to injuries in other brain regions.

Across all brain regions examined, the designation “no injury described” should not be interpreted as indicative of normal histology, particularly when relying solely on standard H&E staining. Subtle microscopic changes may go undetected, and in many cases, neurological impairment is attributable to injury in regions other than the one being assessed. Immunohistochemical markers such as CD68 and GFAP proved valuable in revealing reactive gliosis and microglial activation, offering insight into underlying pathology not captured by conventional methods. Overall, the analysis demonstrated a clear and consistent relationship between increasing histopathologic injury severity and elevated Neurologic Injury Severity (NIS) scores across all six brain regions. This correlation was especially strong in the frontal lobes, deep grey matter, and temporal regions, where CT imaging’s sensitivity to deeper structures likely enhanced detection and alignment with pathology. The trends were most robust in infants, toddlers, and older children, underscoring the reliability of NIS scoring in these age groups. In contrast, findings in neonates were more variable, highlighting the need for refined imaging protocols and further histopathologic validation to optimize NIS as a neuromonitoring tool during ECMO.

## Discussion

A strong correlation was observed between elevated NIS scores and severe histopathologic injury, both globally and regionally, reinforcing the clinical utility of the NIS framework for assessing neurologic burden during ECMO support. These findings suggest that structured imaging-based scoring, using modalities such as CT or head ultrasound, can serve as a meaningful surrogate for underlying neuropathology, particularly in settings where MRI is contraindicated or unavailable. The most robust imaging-pathology concordance was found in the frontal lobes, deep grey matter, and temporal lobes, where subjects with severe injury consistently had NIS scores ≥10. However, the inclusion of neonates through adolescents introduces substantial developmental heterogeneity in cerebral vulnerability, perfusion physiology, and injury patterns. Although age-stratified analyses partially mitigate this limitation, the findings should not be extrapolated uniformly across developmental stages.

These regions may be particularly vulnerable to hypoxic-ischemic injury in pediatric ECMO patients, aligning with prior reports of frontotemporal susceptibility. In contrast, the parieto-occipital lobes, pons, and cerebellum showed more frequent discordance, with several subjects exhibiting elevated NIS scores despite no histopathologic injury described on standard H&E staining. Large focal vascular lesions were readily identified by neuroimaging; however, the clinically relevant uncertainty lies in the detection of diffuse hypoxic-ischemic or microscopic injury, which in some cases was evident only on histopathology or immunohistochemistry. This discrepancy raises two key considerations: first, that the NIS score may reflect localized injury contributing to a high composite score even in the absence of widespread pathology; and second, that conventional histopathologic techniques may miss subtle or subclinical injury, especially in neonates where posterior fossa and brainstem evaluation is technically challenging. Importantly, the label “no injury described” should not be interpreted as an absence of pathology. Immunohistochemical staining with GFAP and CD68 revealed reactive gliosis and microglial activation in several cases, underscoring the limitations of standard H&E staining and the need for more sensitive and regionally targeted neuropathologic approaches.

These findings support further refinement of both imaging protocols and histopathologic validation strategies to enhance the accuracy and reliability of NIS as a neuromonitoring biomarker during ECMO. Additionally, the variability in NIS scores among subjects with severe pathology underscores the heterogeneity and multifactorial nature of brain injury in this population. The observed injuries cannot be attributed specifically to ECMO itself, as hypoxic-ischemic and hemorrhagic injury may arise from antecedent cardiac arrest, systemic hypoperfusion, or primary disease processes. Similarly, differences observed between VV and VA ECMO likely reflect underlying indication and arrest physiology rather than ECMO modality alone. Contributing mechanisms may include altered cerebral autoregulation, fluctuating perfusion pressures, embolic events, and systemic inflammatory responses, particularly in venoarterial ECMO configurations.^22,23^

Emerging studies employing plasma biomarkers and machine learning have further highlighted this complexity by identifying dynamic injury patterns that evolve throughout ECMO support. For instance, machine learning models have been developed to predict brain injury in pediatric ECMO patients, demonstrating the potential of these tools in enhancing diagnostic accuracy and patient management.^24–26^

Our study reinforces the utility of neuroimaging, particularly CT, in detecting significant neurologic injury in pediatric ECMO patients. While MRI is often considered the gold standard for identifying white matter injuries and microhemorrhages due to its superior soft tissue contrast, its application is frequently limited in ECMO settings because of equipment incompatibilities and patient instability.^27^ CT, on the other hand, is more accessible and faster, making it a practical choice in critical care scenarios. Notably, our findings align with previous research indicating that CT has a high positive predictive value for detecting severe brain injuries when performed within 14 days of ECMO initiation.^28^ It’s important to recognize that while MRI offers higher soft tissue resolution, CT provides sufficient detail to identify clinically significant injuries, especially in acute settings. Therefore, CT serves as a reliable diagnostic tool for early detection of severe neurologic injuries in this patient population.

Furthermore, adjunctive neuromonitoring modalities, including amplitude-integrated electroencephalography (aEEG),^29^ commercial cerebral oximeters,^30^ and diffuse optical spectroscopies,^31,32^ have gained traction for their ability to provide real-time cerebral monitoring in ECMO patients. Their incorporation into clinical protocols may enhance the accuracy and timeliness of neurologic assessments during ECMO support. For example, a study by Sanford et al. demonstrated that disruption of cerebral autoregulation, as measured by diffuse correlation spectroscopy, was associated with higher radiographic neurologic injury scores in pediatric ECMO patients.^31^ This study has several limitations. This was a retrospective, single-center, retrospective study with a small imaging-pathology cohort (n=26) which limits statistical power and generalizability. Neuroimaging was obtained up to seven days prior to death; therefore, additional cerebral injury may have occurred between imaging acquisition and neuropathologic examination, potentially contributing to imaging–pathology discordance. Imaging modalities varied across subjects, and CT and head ultrasound lack sensitivity for detecting diffuse, early, or microscopic injury. Although standardized histological sampling was employed, focal pathology may have been missed. Furthermore, combining patients supported with veno-venous ECMO for respiratory failure and veno-arterial ECMO for extracorporeal cardiopulmonary resuscitation introduces substantial physiological heterogeneity and limits mechanistic inference regarding injury patterns. Finally, although imaging and histopathologic assessments were performed in a blinded manner, interobserver variability cannot be excluded; therefore, these findings should be considered exploratory and hypothesis-generating.

## Conclusion

Neuroimaging, particularly CT interpreted through structured severity scoring, correlates strongly with neuropathologic findings in pediatric ECMO decedents. Frontal and deep grey regions demonstrated the clearest alignment, while regional mismatches underscore the need for enhanced histologic methods and adjunctive monitoring. Prospective multicenter studies with standardized imaging protocols and biomarker validation are essential to advance neurologic surveillance in this population.

## Supplementary Material

Supplementary Files

This is a list of supplementary files associated with this preprint. Click to download.

- EquatorSTROBEChecklist.docx
- SupplementaldigitalcontentNCCJ.pdf

## Figures and Tables

**Table 1.** Demographic Characteristics of the Participants

Sample Characteristics	n	%	Mean (μ)	StDev (σ)	Range
**Gender**					
Male	29	53.7			
Female	25	46.3			
**Race**					
White or Caucasian	38	70.4			
Black	15	27.8			
Other/Unknown	1	1.8			
**Ethnicity**					
Hispanic or Latino	15	27.8			
Not Hispanic or Latino	39	72.2			
**Age Category**					
Adolescents	13	24.1			12 – 18 years
Children	15	27.8			4 – 11 years
Infants & Toddlers	17	31.5			29 days – 3 years
Neonates	9	16.6			0 – 28 days
**Primary Diagnoses**					
Pulmonary	24	44.4			
ECPR	21	38.9			
Cardiac	9	16.7			
**Type of ECMO**					
VA ECMO	30	55.6			
VV ECMO	24	44.4			
**Continuous Variables**					
Weight (kg)			20.79	22.63	2.3 – 98.2
Height (cm)			92.69	43.29	44.0 – 180.0

## References

[1] Bulas DI, Taylor GA, O’Donnell RM, Short BL, Fitz CR, Vezina G. Intracranial abnormalities in infants treated with extracorporeal membrane oxygenation: update on sonographic and CT findings. AJNR Am J Neuroradiol 1996;17(2):287–94. (In eng). 8938301 PMC8338373

[2] Organization ELS. ELSO Registry International Summary Report. ELSO. (https://www.elso.org/registry/internationalsummaryandreports/internationalsummary.aspx).

[3] Bembea MM, Felling R, Anton B, Salorio CF, Johnston MV. Neuromonitoring During Extracorporeal Membrane Oxygenation: A Systematic Review of the Literature. Pediatr Crit Care Med 2015;16(6):558–64. (In eng). DOI: 10.1097/PCC.0000000000000415. 25828783

[4] Rilinger JF, Smith CM, deRegnier RAO, Transcranial Doppler Identification of Neurologic Injury during Pediatric Extracorporeal Membrane Oxygenation Therapy. J Stroke Cerebrovasc Dis 2017;26(10):2336–2345. (In eng). DOI: 10.1016/j.jstrokecerebrovasdis.2017.05.022. 28583819

[5] O’Brien NF, Buttram SDW, Maa T, Cerebrovascular Physiology During Pediatric Extracorporeal Membrane Oxygenation: A Multicenter Study Using Transcranial Doppler Ultrasonography. Pediatr Crit Care Med 2019;20(2):178–186. (In eng). DOI: 10.1097/PCC.0000000000001778. 30395027

[6] Lin N, Flibotte J, Licht DJ. Neuromonitoring in the neonatal ECMO patient. Semin Perinatol 2018;42(2):111–121. (In eng). DOI: 10.1053/j.semperi.2017.12.007. 29397959 PMC5875727

[7] Fox J, Jenks CL, Farhat A, EEG is A Predictor of Neuroimaging Abnormalities in Pediatric Extracorporeal Membrane Oxygenation. J Clin Med 2020;9(8) (In eng). DOI: 10.3390/jcm9082512. PMC7463499 32759731

[8] Lazar EL, Abramson SJ, Weinstein S, Stolar CJ. Neuroimaging of brain injury in neonates treated with extracorporeal membrane oxygenation: lessons learned from serial examinations. J Pediatr Surg 1994;29(2):186–90; discussion 190–1. (In eng). DOI: 10.1016/0022-3468(94)90315-8. 8176589

[9] Cho SM, Hwang J, Chiarini G, Neurological monitoring and management for adult extracorporeal membrane oxygenation patients: Extracorporeal Life Support Organization consensus guidelines. Crit Care 2024;28(1):296. (In eng). DOI: 10.1186/s13054-024-05082-z. 39243056 PMC11380208

[10] Hervey-Jumper SL, Annich GM, Yancon AR, Garton HJ, Muraszko KM, Maher CO. Neurological complications of extracorporeal membrane oxygenation in children. J Neurosurg Pediatr 2011;7(4):338–44. (In eng). DOI: 10.3171/2011.1.Peds10443. 21456903

[11] Khanduja S, Kim J, Kang JK, Hypoxic-Ischemic Brain Injury in ECMO: Pathophysiology, Neuromonitoring, and Therapeutic Opportunities. Cells 2023;12(11):1546. (https://www.mdpi.com/2073-4409/12/11/1546). 37296666 10.3390/cells12111546 PMC10252448

[12] Veraar CM, Rinösl H, Kühn K, Non-pulsatile blood flow is associated with enhanced cerebrovascular carbon dioxide reactivity and an attenuated relationship between cerebral blood flow and regional brain oxygenation. Critical Care 2019;23(1):426. DOI: 10.1186/s13054-019-2671-7. 31888721 PMC6937980

[13] Rollins MD, Hubbard A, Zabrocki L, Barnhart DC, Bratton SL. Extracorporeal membrane oxygenation cannulation trends for pediatric respiratory failure and central nervous system injury. J Pediatr Surg 2012;47(1):68–75. (In eng). DOI: 10.1016/j.jpedsurg.2011.10.017. 22244395

[14] Nakip OS, Kesici S, Konuskan GD, Yazici MU, Konuskan B, Bayrakci B. Neurodevelopmental Outcomes of Pediatric Cardiac Extracorporeal Membrane Oxygenation Survivors With Central Cannulation. Am J Intellect Dev Disabil 2024;129(5):377–386. (In eng). DOI: 10.1352/1944-7558-129.5.377. 39197851

[15] Khan IR, Gu Y, George BP, Brain Histopathology of Adult Decedents After Extracorporeal Membrane Oxygenation. Neurology 2021;96(9):e1278–e1289. (In eng). DOI: 10.1212/wnl.0000000000011525. 33472914 PMC8055323

[16] Aludaat C, Sarsam M, Doguet F, Baste JM. Autopsy and clinical discrepancies in patients undergoing extracorporeal membrane oxygenation: a case series-a step towards understanding “Why”? J Thorac Dis 2019;11(Suppl 15):S1865–s1868. (In eng). DOI: 10.21037/jtd.2019.08.121. 31632770 PMC6783783

[17] Wien MA, Whitehead MT, Bulas D, Patterns of Brain Injury in Newborns Treated with Extracorporeal Membrane Oxygenation. AJNR Am J Neuroradiol 2017;38(4):820–826. (In eng). DOI: 10.3174/ajnr.A5092. 28209579 PMC7960244

[18] Zhang H, Xu J, Yang X, Narrative Review of Neurologic Complications in Adults on ECMO: Prevalence, Risks, Outcomes, and Prevention Strategies. Front Med (Lausanne) 2021;8:713333. (In eng). DOI: 10.3389/fmed.2021.713333. 34660625 PMC8513760

[19] Taylor GA, Fitz CR, Glass P, Short BL. CT of cerebrovascular injury after neonatal extracorporeal membrane oxygenation: implications for neurodevelopmental outcome. AJR Am J Roentgenol 1989;153(1):121–6. (In eng). DOI: 10.2214/ajr.153.1.121. 2660530

[20] Taylor GA, Fitz CR, Miller MK, Garin DB, Catena LM, Short BL. Intracranial abnormalities in infants treated with extracorporeal membrane oxygenation: imaging with US and CT. Radiology 1987;165(3):675–8. (In eng). DOI: 10.1148/radiology.165.3.3317499. 3317499

[21] Farhat A, Li X, Huet B, Tweed J, Morriss MC, Raman L. Routine Neuroimaging: Understanding Brain Injury in Pediatric Extracorporeal Membrane Oxygenation. Crit Care Med 2022;50(3):480–490. (In eng). DOI: 10.1097/CCM.0000000000005308. 34637418

[22] Polito A, Barrett CS, Wypij D, Neurologic complications in neonates supported with extracorporeal membrane oxygenation. An analysis of ELSO registry data. Intensive Care Med 2013;39(9):1594–601. (In eng). DOI: 10.1007/s00134-013-2985-x. 23749154

[23] Tian F, Morriss MC, Chalak L, Impairment of cerebral autoregulation in pediatric extracorporeal membrane oxygenation associated with neuroimaging abnormalities. Neurophotonics 2017;4(4):041410. (10.1117/1.NPh.4.4.041410). 28840161 PMC5562949

[24] Hong SJ, De Souza BJ, Penberthy KK, Plasma brain-related biomarkers and potential therapeutic targets in pediatric ECMO. Neurotherapeutics 2025;22(1):e00521. (In eng). DOI: 10.1016/j.neurot.2024.e00521. 39765416 PMC11840354

[25] Bembea MM, Savage W, Strouse JJ, Glial fibrillary acidic protein as a brain injury biomarker in children undergoing extracorporeal membrane oxygenation. Pediatr Crit Care Med 2011;12(5):572–9. (In eng). DOI: 10.1097/PCC.0b013e3181fe3ec7. 21057367 PMC3686089

[26] Deng B, Zhao Z, Ruan T, Development and external validation of a machine learning model for brain injury in pediatric patients on extracorporeal membrane oxygenation. Crit Care 2025;29(1):17. (In eng). DOI: 10.1186/s13054-024-05248-9. 39789565 PMC11716487

[27] Sabir H, Kipfmueller F, Bagci S, Feasibility of bedside portable MRI in neonates and children during ECLS. Crit Care 2023;27(1):134. (In eng). DOI: 10.1186/s13054-023-04416-7. 37016432 PMC10071221

[28] Custer C, Singh S, Sanford E, Computed Tomography Is Predictive of Significant Neurologic Injury in Children Supported on Extracorporeal Membrane Oxygenation. ASAIO Journal 2023;69(11):e460–e462. DOI: 10.1097/mat.0000000000001990. 37314831 PMC11834951

[29] Chahine A, Chenouard A, Joram N, Continuous Amplitude-Integrated Electroencephalography During Neonatal and Pediatric Extracorporeal Membrane Oxygenation. J Clin Neurophysiol 2023;40(4):317–324. (In eng). DOI: 10.1097/WNP.0000000000000890. 34387276

[30] Zhang M, Yang Y, Chen X, Application of Near-Infrared Spectroscopy to Monitor Perfusion During Extracorporeal Membrane Oxygenation After Pediatric Heart Surgery. Front Med (Lausanne) 2021;8:762731. (In eng). DOI: 10.3389/fmed.2021.762731. 34881265 PMC8645544

[31] SanfordEL, AkoredeR, MillerI, Association Between Disrupted Cerebral Autoregulation and Radiographic Neurologic Injury for Children on Extracorporeal Membrane Oxygenation: A Prospective Pilot Study. ASAIO J 2023;69(7):e315–e321. (In eng). DOI: 10.1097/MAT.0000000000001970.37172001

[32] Busch DR, Baker WB, Mavroudis CD, Noninvasive optical measurement of microvascular cerebral hemodynamics and autoregulation in the neonatal ECMO patient. Pediatr Res 2020;88(6):925–933. (In eng). DOI: 10.1038/s41390-020-0841-6. 32172282 PMC7492409

